# Genomic characterization and phylogenetic analysis of chloroplast genome of *Eurya nitida* (*Eurya* Thunberg)

**DOI:** 10.1080/23802359.2025.2505249

**Published:** 2025-05-15

**Authors:** Yuyu Han, Dongzhen Jiang, Lei Zhou, Tao Jiang

**Affiliations:** College of Forestry, Guizhou University, Guiyang, China

**Keywords:** *Eurya nitida*, chloroplast genome, phylogenetic analysis

## Abstract

This study aimed to characterize the chloroplast genome of *Eurya nitida* Korthals. The complete chloroplast genome of *Eurya nitida* was sequenced and assembled using high-throughput sequencing technology. The genome spans 157,191 bp and exhibits a typical quadripartite structure, comprising a large single-copy region (LSC, 87,231 bp), a small single-copy region (SSC, 18,215 bp), and two inverted repeat regions (IRs, 51,744 bp each). Phylogenetic analysis revealed a close relationship between *E. nitida* and *Eurya hebeclados* Ling. This study elucidates the structural features and phylogenetic position of the *E. nitida* chloroplast genome.

## 1. Introduction

*Eurya nitida* Korthals, first described by Korthals in 1840, belongs to the genus *Eurya* Thunberg. It’s taxonomic classification was historically ambiguous due to morphological similarities with related species (Haan and Korthals 1840). Advances in plant taxonomy during the 19th and 20th centuries clarified its distinction through morphological traits such as leaf shape and inflorescence structure (Ling 1951; Qiu and Zhong 1987). By the mid-twentieth century, *E. nitida* was definitively classified within the Pentaphylacaceae family, with boundaries delineated *via* morphological, ecological, and genetic comparisons against species like *Eurya loquaiana* Dunn and *E. hebeclados* Ling (Huang et al. 2004; Shi et al. 2008, 2009, 2012, 2014). Currently, *E. nitida* is recognized as a distinct species, primarily differentiated by shoot and leaf morphology. Chloroplast genomes have become pivotal in resolving taxonomic ambiguities and phylogenetic relationships, particularly in complex taxa (Ran et al. 2024; Xiao et al. 2024). However, the absence of chloroplast genomic data for *E. nitida* has hindered its comprehensive study. This research addresses this gap by characterizing the *E. nitida* chloroplast genome, thereby facilitating resource development, phylogenetic reconstruction, and evolutionary classification within *Eurya* Thunberg.

## 2. Materials and methods

### 2.1. Sample collection and DNA extraction

**Figure 1.**
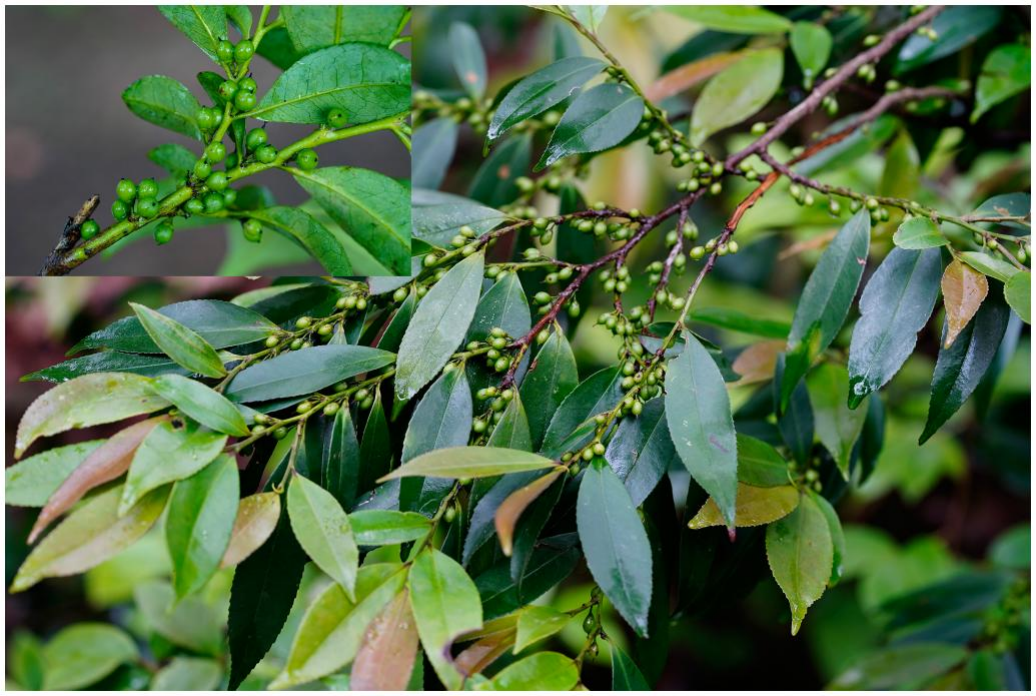
Reference image of *Eurya nitida*. (this photograph was taken by the authors of this article, Dongzhen Jiang and Lei Zhou. Leaves thinly leathery, elliptic, oblong-elliptic, margins densely serrate or finely obtusely toothed; fruit orbicular, blue-black at maturity.).

Fresh leaves of *E. nitida* were collected from the Maolan National Nature Reserve (25.22015921 N, 107.92730734 E), Guizhou Province, China (Figure 1). Voucher specimens (Contact Person: Tao Jiang, tjiang5@gzu.edu.cn, voucher number JT-20241111) were deposited at the Tree Specimen Laboratory, College of Forestry, Guizhou University. Chloroplast DNA was extracted using a modified CTAB protocol (Pahlich and Gerlitz 1980). DNA integrity was assessed *via* 1% agarose gel electrophoresis, while purity and concentration were measured using a NanoDrop spectrophotometer.

### 2.2. Sequencing and genome assembly and repeat sequence analysis

Sequencing libraries were prepared through fragmentation, end repair, and adapter ligation. Libraries were sequenced on the Illumina NovaSeq 6000 platform. Raw reads were assembled de novo using GetOrganelle v.1.7.5.3 (Jin et al. 2020), followed by annotation with CPGAVAS2 (Shi et al. 2019). The complete chloroplast genome was deposited in NCBI (Accession: PQ685675) and visualized using OGDRAW (Lohse et al. 2007). In this study, simple sequence repeats (SSRs) within the *E. nitida* chloroplast genome were analyzed using MISA v.2.1 software (Beier et al. 2017). The detection parameters for repeat units were defined as follows: mononucleotides (≥10 repeats), dinucleotides (≥5 repeats), trinucleotides (≥4 repeats), tetranucleotides (≥3 repeats), pentanucleotides (≥3 repeats), and hexanucleotides (≥6 repeats). For larger repeat sequences, the REPuter online tool (Kurtz et al. 2001) was employed with a Hamming distance of 3 and a minimum repeat size of 30 bp. Four repeat types were identified: forward (F), reverse (R), complement (C), and palindromic (P).

### 2.3. Phylogenetic analysis

Chloroplast genomes of 24 species (15 *Eurya* species and and the *Apterosperma oblata* (Accession: NC035641) was used as an outgroup) were retrieved from National Center for Biotechnology Information (NCBI). Sequences were aligned using MAFFT 7 (Katoh and Standley 2014), and MEGAX (Kumar et al. 2018) was used to manually correct and select the best maximum likelihood (ML) method to construct the phylogenetic tree model (GTR+I + G). The phylogenetic tree was constructed in IQ-TREE v.2.2.0, and the self-expansion support was set to 1000 (Trifinopoulos et al. 2016). The optimal model (HKY+G + I) was identified using MrModeltest v.2.3, and a Bayesian inference (BI) phylogenetic tree was subsequently reconstructed using MrBayes v.3.2.7 (Huelsenbeck and Ronquist 2001). Finally the phylogenetic tree was landscaped using the online tool iTOL V4 (Letunic and Bork 2021).

## 3. Results

### 3.1. Chloroplast genome structure

**Figure 2.**
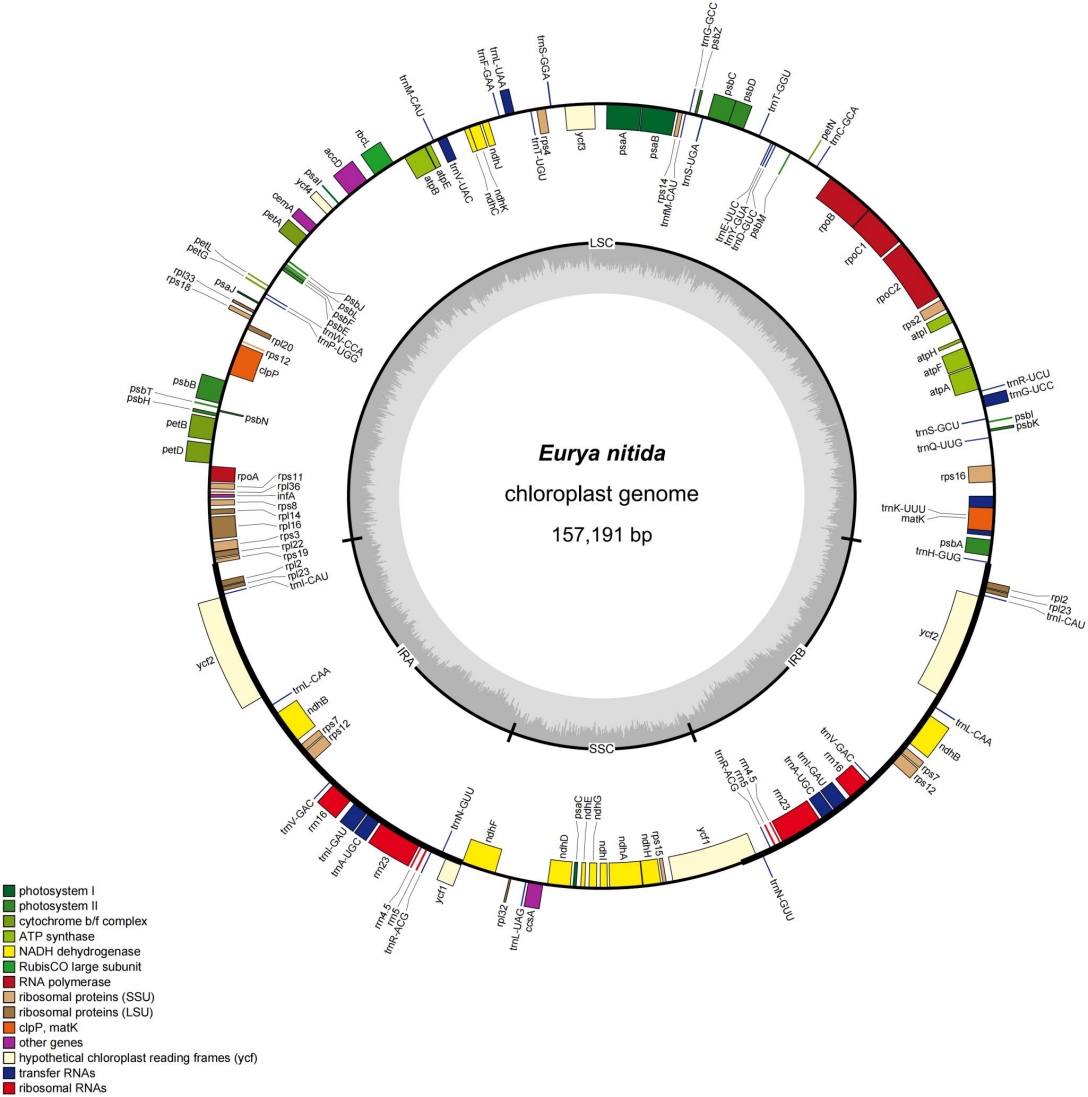
Chloroplast genome map of *Eurya nitida*. (Genes shown inside the circle are transcribed clockwise, those outside the circle are counterclockwise transcribed. The light grey and the darker grey in the inner circle represent AT and GC content, respectively. Different functional groups of genes are signed according to the colored boxes. LSC: large single copy; SSC: small single copy; IRA/IRB: Inverted repeat regions.).

The minimum and average read mapping depths were 367× and 1548.7×, respectively (Figure S1). Meanwhile, the structures of the trans-splicing and cis-splicing genes are shown in Figures S2 and S3. The *E. nitida* chloroplast genome (157,191 bp) comprises an LSC (87,231 bp), SSC (18,215 bp), and two IRs (51,744 bp each) (Figure 2). The GC content was 37.34% overall, varying regionally: LSC (35.32%), SSC (31.04%), and IRs (42.98%). The genome harbors 130 genes (85 protein-coding, 37 tRNA, 8 rRNA) (Table S1).

**Figure 3.**
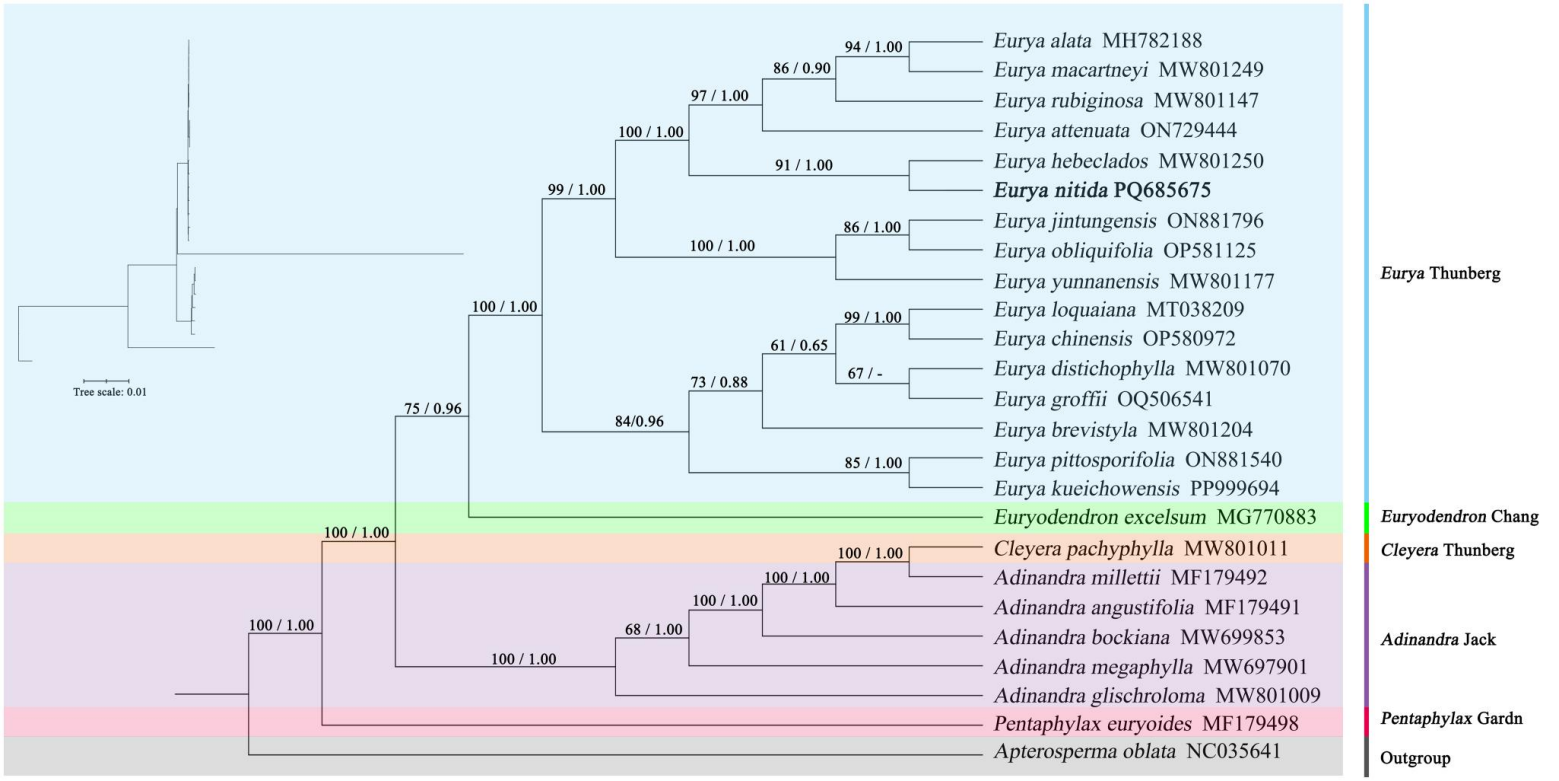
Phylogenetic tree obtained *via* the maximum likelihood (ML) and bayesian inference (BI) methods of 25 species on the basis of complete cp genomes. (outgroup: *Apterosperma oblata* (NC035641); maximum likelihood (ML) and bayesian (BI) trees, BS ≥ 50% and PP ≥ 0.95 are indicated above branches as BS/PP) The following sequences, of which some already existed in NCBI database but were unpublished, were used: *Eurya alata* MH782188 (Zhang et al. 2021), *Eurya brevistyla* MW801204, *Eurya chinensis* OP580972, *Eurya distichophylla* MW801070, *Eurya groffii* OQ506541, *Eurya hebeclados* MW801250, *Eurya jintungensis* ON881796, *Eurya kueichowensis* PP999694, *Eurya loquaiana* MT038209 (Wang et al. 2020), *Eurya macartneyi* MW801249, *Eurya obliquifolia* OP581125, *Eurya pittosporifolia* ON881540, *Eurya rubiginosa* MW801147, *Eurya attenuata* ON729444 (Li et al. 2023), *pentaphylax euryoides* MF179498, *Adinandra glischroloma* MW801009, *Adinandra bockiana* MW699853 (Nguyen et al. 2023), *Adinandra megaphylla* MW697901, *Adinandra millettii* MF179492, *Euryodendron excelsum* MG770883 (Shi et al. 2019), *Cleyera pachyphylla* MW801011, *Adinandra angustifolia* MF179491, *Apterosperma oblata* NC035641.

### 3.2. Repeat sequence analysis

A total of 209 SSRs were identified, predominantly mononucleotide repeats (127), followed by tri- (72), tetra- (6), di- (3), and pentanucleotide (1) repeats (Figure S4A). SSRs were enriched in the LSC (62.7%) and sparse in IRs (18.2%) (Figure S4B, C). Long repeats included forward (21), palindromic (27), and reverse (2) types (Figure S4D).

### 3.3. Phylogenetic analysis

In this study, phylogenetic analysis (Figure 3) resolved *E. nitida* as a member of *Eurya* Thunberg, forming a clade with *E. hebeclados* (ML bootstrap = 91, BI posterior probability = 1.00). Sister branches included *E. alata*, *E. macartneyi*, *E. rubiginosa*, and *E. attenuata*.

## 4. Discussion

Chloroplast genomes are highly conserved in structure and maternally inherited, making them ideal for phylogenetic studies (Chen et al. 2010; Zou et al. 2021). The *E. nitida* genome aligns with reported *Eurya* chloroplast genomes (150–160 kb, quadripartite structure) and exhibits conserved GC content trends (Wang et al. 2020).

In this study, the whole genome sequence of *E. nitida* chloroplast was included in the NCBI database, which added new content to the *Eurya* Thunberg chloroplast genome data. Simple repeat sequences (SSRs) are commonly used in plant identification and genetic mapping because of their high content and polymorphism (Du et al. 2012). In this study, we found that the chloroplast genome of *E. nitida* contains 209 SSR sites, mainly single nucleotide repeats, with no C-type long repeats, and mainly polyA and polyT, which provides a basis for genetic analysis (Kuang et al. 2011). Chloroplast genome sequences can be used to infer phylogenetic relationships and determine species affinities (Xue et al. 2021). The present study supports that *E. nitida* is a member of *Eurya* Thunberg and obtains a high support rate; it also further finds its closest related species, *E. hebeclados*, and its sister branches. This is a crucial step in exploring the molecular phylogenetic relationships of *E. nitida*, which will help the study of molecular systematics and population evolution of *Eurya* Thunberg plants.

## 5. Conclusions

This study presents the first complete chloroplast genome of *E. nitida*, revealing its structural features, repeat content, and phylogenetic position. The data provide a foundation for future studies on *Eurya* taxonomy, evolution, and conservation.

## Supplementary Material

Supplementary Figure.docx

Supplementary Table S1.xls

## Data Availability

The complete chloroplast genome sequence of *Eurya nitida* has been submitted to the GenBank database. The accession number for this sequence is PQ685675, which is automatically generated by the NCBI. In addition, the associated BioProject number is PRJNA1196651, the SRA accession number is SRR31703878, and the Bio-Sample number is SAMN45605227.
