# Supplementary material for: Genomic characterization and phylogenetic analysis of chloroplast genome of Eurya nitida (Eurya Thunberg)
Source: Mitochondrial DNA B Resour. 2025 May 15;10(6):480–4. doi: 10.1080/23802359.2025.2505249 (PMC12082727; doi:10.1080/23802359.2025.2505249)
Supplement: Supplementary Figure.docx [file TMDN_A_2505249_SM8930.docx]

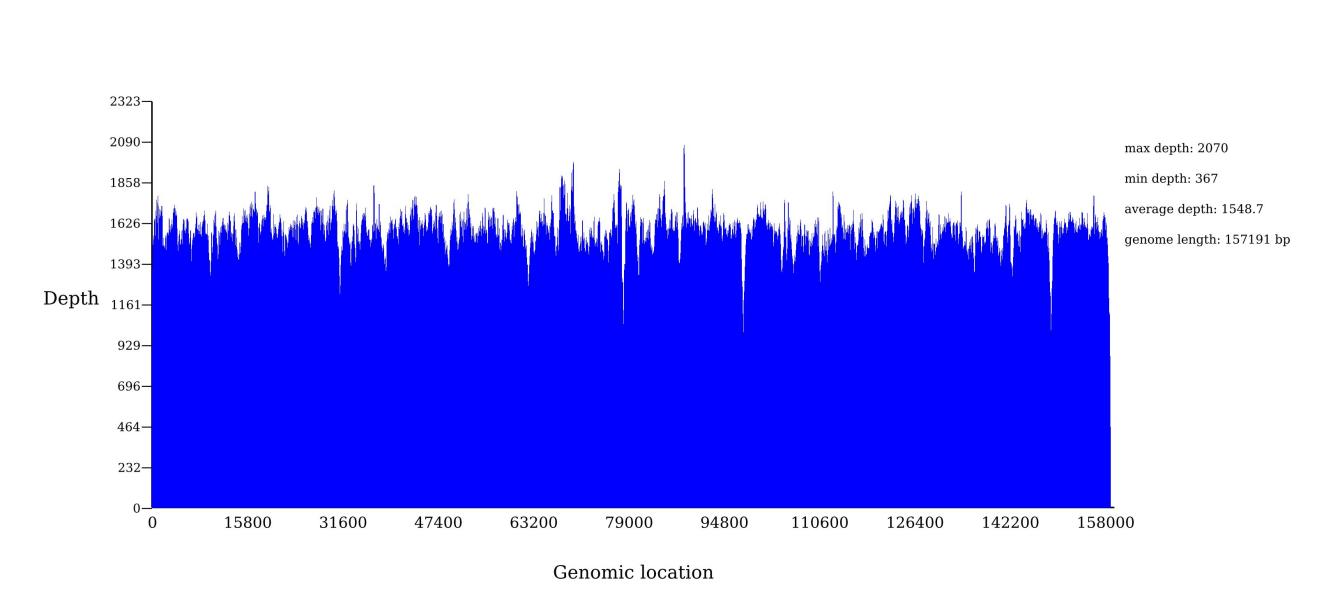


Figure S1. Sequencing depth distribution of the chloroplast genome of *Eurya nitida*. (The figure shows the sequencing depth distribution of the chloroplast genome of the species, with the horizontal axis indicating the genomic location and the vertical axis indicating the sequencing depth.)


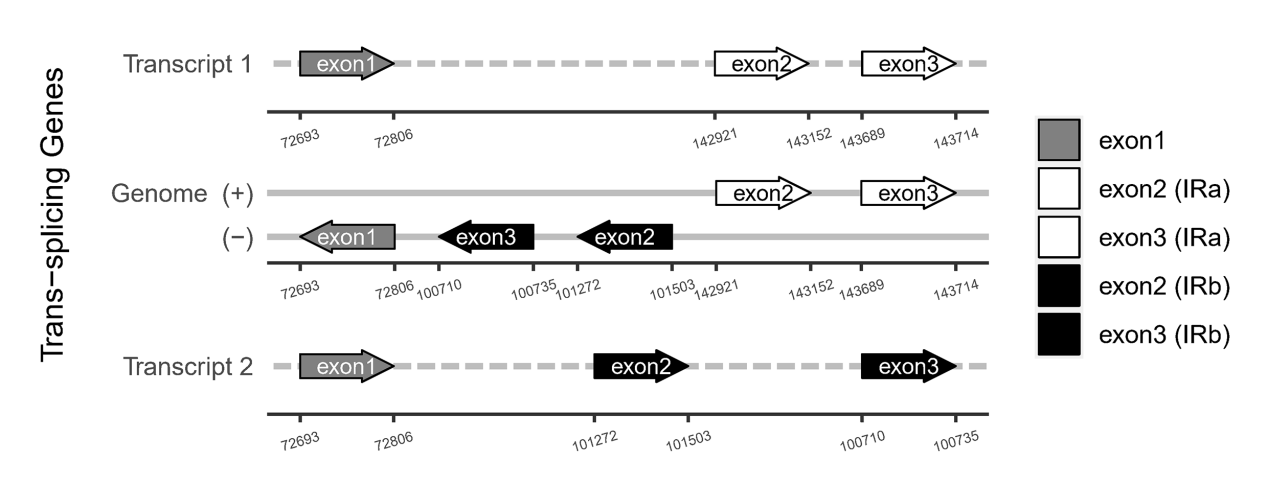


Figure S2. Schematic representation of the trans-splicing genes in the chloroplast genome of *Eurya nitida* analyzed using CPGView.


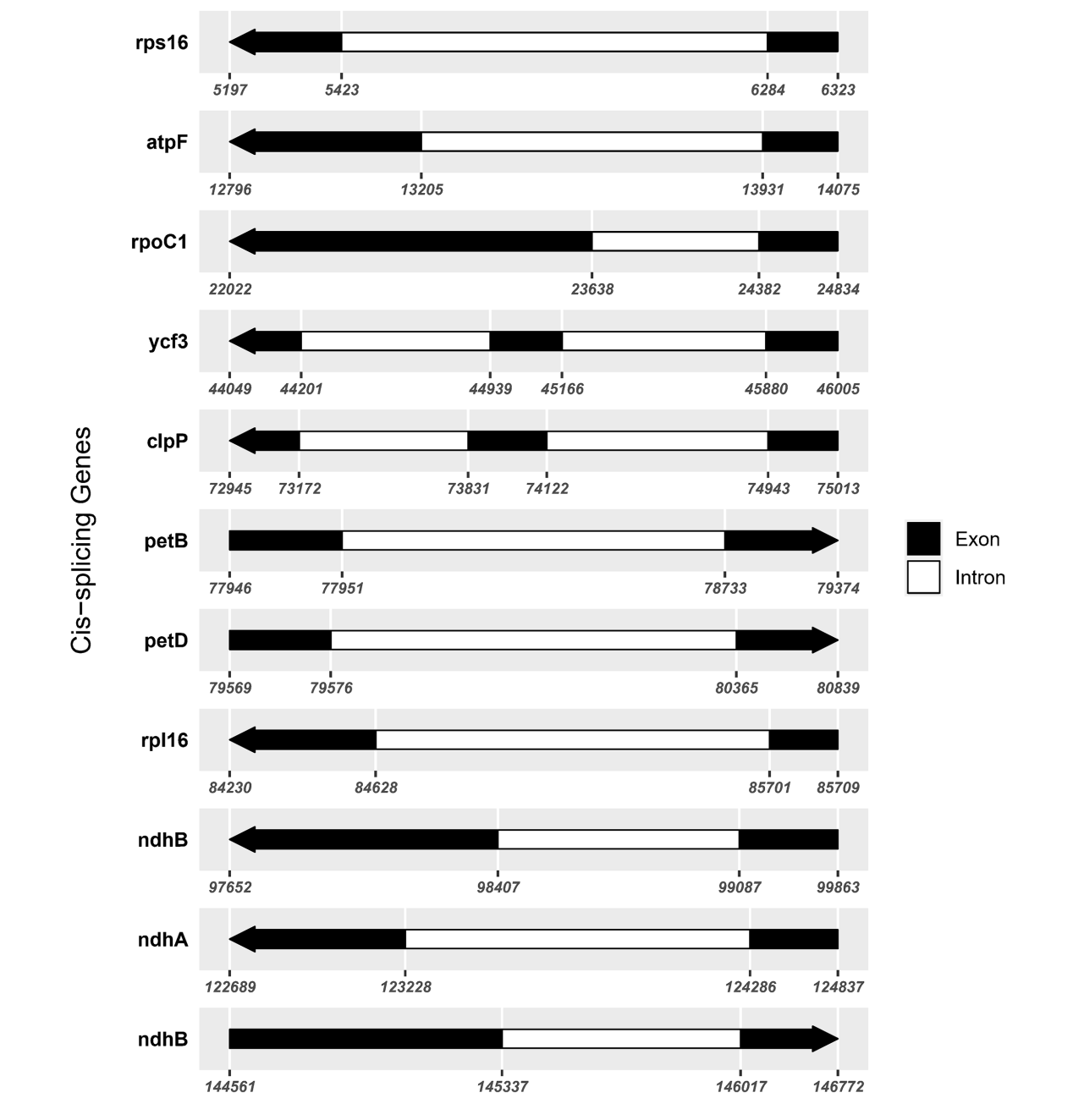


Figure S3. Structure of cis-splicing genes in the *Eurya nitida* cp genome. (Exons of the cis-splicing gene are shown in black; introns are indicated in white. Arrows indicate the sense direction of the gene. Note that the lengths of the exons and introns are not drawn to scale.)


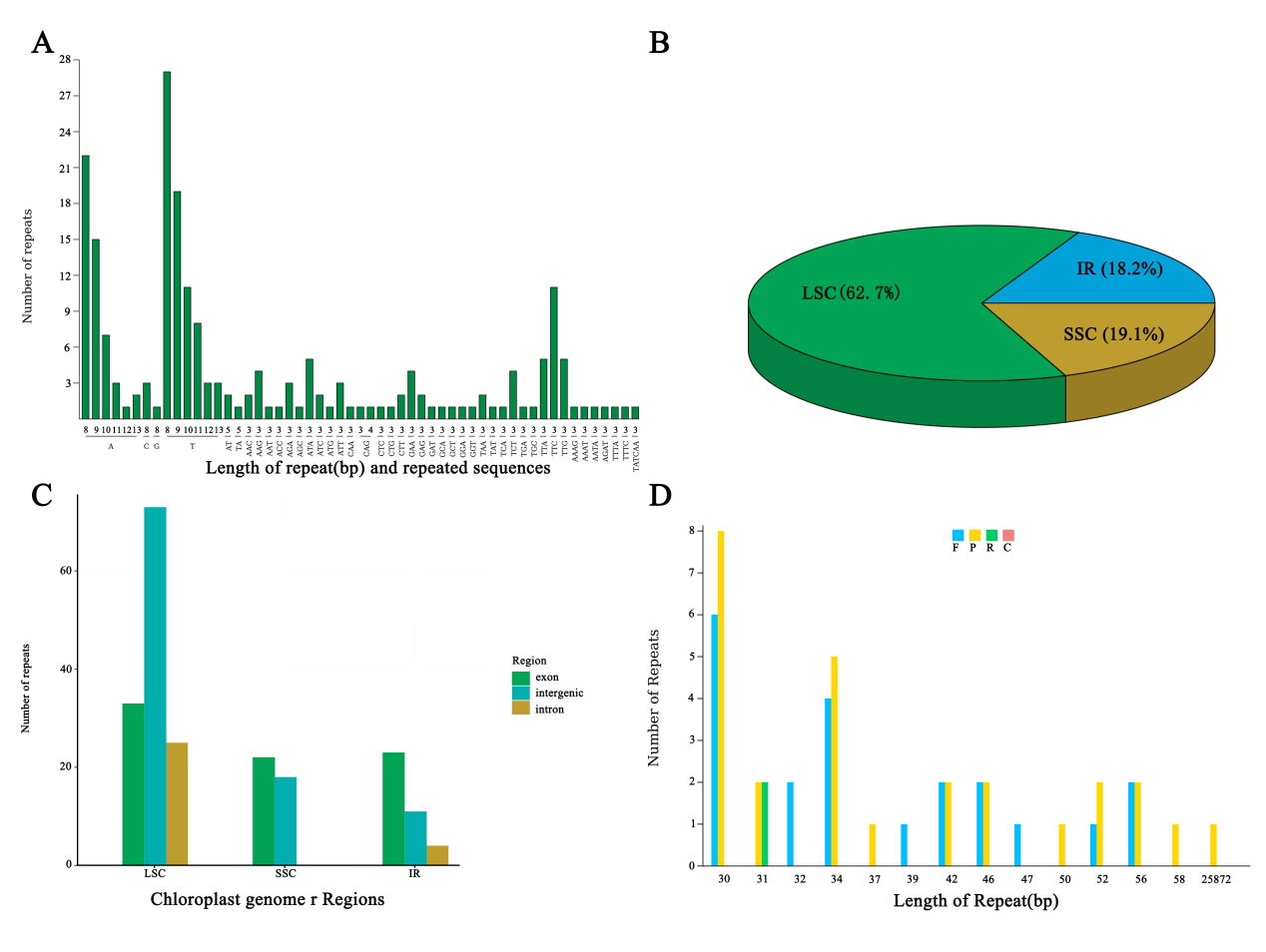


Figure S4. Repeat sequence analysis of the chloroplast genomes of the *Eurya nitida*. (A: Number of SSR repetitive sequence types; B: Proportion of SSRs in the different regions; C: The number of SSRs in the different regions; D: Number of four dispersed repetitive sequence types.)
